# Low-dose anti-thymocyte globulin plus low-dose post-transplant cyclophosphamide-based regimen for prevention of graft-versus-host disease after haploidentical peripheral blood stem cell transplants: a large sample, long-term follow-up retrospective study

**DOI:** 10.3389/fimmu.2023.1252879

**Published:** 2023-10-26

**Authors:** Xingying Li, Jun Yang, Yu Cai, Chongmei Huang, Xiaowei Xu, Huiying Qiu, Jiahua Niu, Kun Zhou, Ying Zhang, Xinxin Xia, Yu Wei, Chang Shen, Yin Tong, Baoxia Dong, Liping Wan, Xianmin Song

**Affiliations:** ^1^ Department of Hematology, Shanghai General Hospital, Shanghai Jiaotong University School of Medicine, Shanghai, China; ^2^ Engineering Technology Research Center of Cell Therapy and Clinical Translation, Shanghai Science and Technology Committee (STCSM), Shanghai, China

**Keywords:** graft-versus-host disease, haploidentical, peripheral blood stem cell transplantation, anti-thymocyte globulin, cyclophosphamide

## Abstract

**Introduction:**

The novel low-dose anti-thymocyte (ATG, 5 mg/kg) plus low-dose post-transplant cyclophosphamide (PTCy, 50 mg/kg) (low-dose ATG/PTCy)-based regimen had promising activity for prevention of graft-versus-host disease (GVHD) in haploidentical-peripheral blood stem cell transplantation (haplo-PBSCT), but its impacts on long-term outcomes remain to be defined.

**Methods:**

We performed a large sample, long-term follow-up retrospective study to evaluate its efficacy for GVHD prophylaxis.

**Results:**

The study enrolled 260 patients, including 162 with myeloid malignancies and 98 with lymphoid malignancies. The median follow-up time was 27.0 months. For the entire cohort, the cumulative incidences (CIs) of grade II-IV and III-IV acute GVHD (aGVHD) by 180 days were 13.46% (95% CI, 9.64%-17.92%) and 5.77% (95% CI, 3.37%-9.07%); while total and moderate/severe chronic GVHD (cGVHD) by 2 years were 30.97% (95% CI, 25.43%-36.66%) and 18.08% (95% CI, 13.68%-22.98%), respectively. The 2-year overall survival (OS), relapse-free survival (RFS), GVHD-free, relapse-free survival (GRFS), non-relapse mortality (NRM), and CIs of relapse were 60.7% (95% CI, 54.8%-67.10%), 58.1% (95% CI, 52.2%-64.5%), 50.6% (95% CI, 44.8-57.1%), 23.04% (95% CI, 18.06%-28.40%), and 18.09% (95% CI, 14.33%-23.97%, respectively. The 1-year CIs of cytomegalovirus (CMV) and Epstein–Barr virus (EBV) reactivation were 43.46% (95% CI, 37.39%-49.37%) and 18.08% (95% CI, 13.68%-22.98%), respectively. In multivariate analysis, the disease status at transplantation was associated with inferior survivor outcomes for all patients and myeloid and lymphoid malignancies, while cGVHD had superior outcomes for all patients and myeloid malignancies, but not for lymphoid malignancies.

**Discussion:**

The results demonstrated that the novel regimen could effectively prevent the occurrence of aGVHD in haplo-PBSCT.

## 1 Introduction

Graft-versus-host disease (GVHD) is still a significant barrier to the survival of patients who undergo haploidentical hematopoietic stem cell transplantation (haplo-HSCT) (1, 2). T cell depletion (TCD) of the grafts *ex vivo* and *in vivo* is the main strategy for GVHD prophylaxis. Because the removal of T cells from the graft *ex vivo* significantly increases the risk of graft failure, infection, and disease recurrence, the *in vivo* T-cell removal strategy is more commonly used (3–5). Two kinds of *in vivo* TCD strategies are widely used in haplo-HSCT, including anti-thymocyte globulin (ATG, Thymoglobin^®^, Genzyme Polyclonals S.A.S)-based (6) and post-transplant cyclophosphamide (PTCy)-based regimens (7, 8). Clinical studies have shown the efficacy of ATG in preventing GVHD after allogeneic HSCT for a variety of diseases (9–14). The granulocyte colony-stimulating factor (G-CSF)/ATG-based Beijing protocol was one of the most commonly used regimens after haplo-HSCT because it has a stronger graft-versus-leukemia (GVL) effect among certain patients at high risk of relapse, with outcomes at least comparable to HLA-matched sibling donor transplantation (MSDT) (15). However, it has been found to have relatively high incidences of grade II-IV acute GVHD (aGVHD) with 33.4%-46%, and 12%-14.9% for grade III-IV (16). Due to a slower immune reconstitution, the risk of viral reactivation was increased with the 100-day cumulative incidences (CIs) of cytomegalovirus (CMV) and Epstein–Barr virus (EBV) viremia of over 60% (6, 17) and 50% (17–20), respectively. The post-transplant cyclophosphamide (PTCy)-based Baltimore protocol has also made great advances. A high dose of PTCy substantially mitigates alloreactivity after haploidentical bone marrow transplantation (haplo-BMT), to the point that outcomes are equivalent to patients undergoing HLA-matched donor transplantation (7). It had outstanding outcomes for GVHD prevention with an incidence rate of 21%-32% for grade II-IV aGVHD in haplo-BMT (21, 22); while, by substituting bone marrow (BM) grafts with peripheral blood stem cell (PBSC) grafts, the efficacy of GVHD prophylaxis was decreased with higher incidences of grade II–IV aGVHD (38%-42%) (21, 23, 24). To improve the efficacy of GVHD prophylaxis for haploidentical peripheral blood stem cell transplantation (haplo-PBSCT), the combination of ATG and PTCy has been documented in several reports with reduced incidences of GVHD and acceptable relapse rates (25–28). We have developed a novel regimen of low-dose ATG (5 mg/kg) plus low-dose PTCy (50 mg/kg) combined with cyclosporine (CsA) and mycophenolate mofetil (MMF) (low-dose ATG/PTCy-based) for GVHD prophylaxis in haplo-PBSCT and our previous studies with small sample size and short-time follow-up indicated that the low-dose ATG/PTCy-based regimen had promising activity for GVHD prophylaxis in haplo-PBSCT with CIs for grade II–IV aGVHD of 15.20%-19.40% (29–31). To confirm the efficacy of the low-dose ATG/PTCy-based regimen, a retrospective study with a large sample and long-term follow-up was performed.

## 2 Materials and methods

### 2.1 Patients

A retrospective study was performed for adult patients with hematologic malignancies who underwent haplo-PBSCT in our center from May 2017 to December 2021. All the patients received the low-dose ATG/PTCy-based regimen for GVHD prophylaxis. The graft source was from mobilized PBSCs with G-CSF. A single of unrelated cord blood cells was prescribed as the third-party cells for a minority of patients. Family members selected as haploidentical donors were defined on human leukocyte antigen (HLA)-A, -B, -C, -DRB1, and -DQB1 locus at the high-resolution level with the recipient -donor mismatched number (HLA) ≥3 (21). The study had ethical approval from hospital ethical committees (No: 2022KY023) and was conducted in accordance with the Declaration of Helsinki. All patients included in the study signed informed consent.

### 2.2 Conditioning regimens and GVHD prophylaxis

Reduced-intensity conditioning (RIC) regimens were prescribed for patients with advanced age (≥55 years) or hematopoietic cell transplantation- comorbidity index (HCT-CI) above 2, while myeloablative conditioning (MAC) regimens were designed for other patients (**Supplementary Figure 1**) (32). For myeloid malignancies, the MAC regimen was composed of intravenous busulfan (Bu, 3.2 mg/kg/day for 4 days), fludarabine (Flu, 30 mg/m^2^/day), and cytarabine (Ara-C, 1-2 g/m^2^/day) both for 5 days; while the RIC regimen included Bu (3.2 mg/kg/d for 2 days), Flu and Ara-C for 5 days with the same doses as in the MAC regimen, total body irradiation (TBI, 3Gy on the day -1). For lymphoid malignancies, the MAC regimens included TBI-based and Bu-based regimens. The TBI-based regimen was composed of 10Gy fractioned TBI (FTBI), cyclophosphamide (Cy, 50 mg/kg/d for 2 days), and etoposide (VP-16, 10 mg/kg/d for 2 days); while Bu-based regimen consisted of Bu (3.2 mg/kg/d for 4 days) combined with the same doses of Cy and VP-16 as above. The RIC regimen included intravenous Bu (3.2 mg/kg/d for 2 days), Cy and VP16 with the same doses as in the MAC regimen, and TBI (3Gy).

All the patients received the low-dose ATG/PTCy-based regimen for prophylaxis of GVHD including ATG 2.5 mg/kg/d on day -2 to -1, Cy 50 mg/kg/d on day +3, CsA and MMF initiating on day +4. The starting infusion dose of CsA was 2 mg/kg, after which the dose was modified to obtain a nadir serum level between 200 and 300 ng/ml, eventually tapering from day +90 to day +180. MMF was administered orally at 15 mg/kg three times daily (maximum dose of 3 g per day) until day +34 and discontinued if no aGVHD (29).

### 2.3 Supportive care

G-CSF was given to all patients starting on day +5 at 5 µg/kg/day until neutrophil recovery. Prophylactic ganciclovir at 5 mg/kg/12h was given to patients during the conditioning period for 1 week. Prophylactic antifungals were used from conditioning until at least 3 months after transplantation. CMV-DNA in serum and EBV-DNA in whole blood were routinely monitored by quantitative polymerase chain reaction once a week until at least day +100.

### 2.4 Definitions

Neutrophil engraftment was defined as obtaining an absolute neutrocyte (ANC) ≥ 0.5 × 10^9^/L for 3 consecutive days after transplantation without G-CSF. Platelet engraftment was defined as obtaining a platelet count ≥ 20 × 10^9^/L for the first of 7 consecutive days without platelet transfusion (33). Full donor chimerism was defined as ≥ 95% of donor T cells in BM samples (34). Graft failure was defined as failure of neutrophil engraftment on day 28 following transplantation (primary graft failure, PGF), or loss of donor chimerism after initial engraftment at any time without disease relapse (secondary graft failure, SGF) (33). aGVHD was diagnosed and graded in line with the modified Glucksberg criteria (35), and chronic GVHD (cGHVD) according to the 2014 National Institutes of Health consensus criteria (36). Morphologic complete remission (CR) was determined by the International Working Group (IWG) and National Comprehensive Cancer Network (NCCN)guidelines (version 3.2013) criteria (37–40) and patients not in morphologic CR were considered to have an active disease (41). Relapse was defined by the appearance of blasts in the peripheral blood (PB) or BM (>5%) after CR (21). Non-relapse mortality (NRM) was defined as death from any cause other than relapse. Overall survival (OS) was defined as the time from the day of stem cell infusion to death from any cause or follow-up. Relapse-free survival (RFS) was defined as survival in continuous CR. GVHD-free and relapse-free survival (GRFS) was defined as survival without the following events: grade III-IV aGVHD, severe cGVHD, disease relapse, or death from any cause after haplo-HSCT (42).

### 2.5 Statistical analysis

The main endpoints of this study included the CIs of aGVHD, cGVHD, relapse, and NRM, and the probabilities of OS, RFS, and GRFS. Survival curves were plotted using the Kaplan-Meier method, and subgroups were compared by log-rank tests. Relapse, NRM, and GVHD were calculated using a CI estimate to accommodate the following competing events (death for relapse, relapse for NRM, and both death and relapse for GVHD), and subgroups were compared by the Fine and Gray test. The prognostic significances of covariates affecting OS, RFS, and GRFS were determined by the Cox proportional hazards regression model. The prognostic significances of covariates affecting the CIs of relapse, NRM, and GVHD were determined using Fine-Gray proportional hazards regression for competing events. Multivariate analyses were performed using variables with a P value < 0.10 in prior univariate analyses. Continuous variables and percentages for categorical variables were expressed via median values and ranges. The Mann-Whitney test was used to analyze continuous variables. All statistical analyses were performed using ‘R’ software version 4.2.1. Statistical significance was set at P value < 0.05.

## 3 Results

### 3.1 Patient and donor characteristics

A total of 260 patients were enrolled in the study. Details of the patient, donor, and allograft characteristics are summarized in **Table 1**. In total, 162 patients with myeloid malignancies were enrolled in the study, including 130 with acute myeloid leukemia (AML), 6 with chronic myelomonocytic leukemia (CMML), and 26 with myelodysplastic syndrome (MDS), while 98 patients with lymphoid malignancies were enrolled, including 62 with acute lymphoblastic leukemia (ALL), 35 with non-Hodgkin’s lymphoma (NHL), and 1 with multiple myeloma (MM). The last enrolled patient underwent HSCT at least 12 months before the initiation of follow-up. The median follow-up time was 27.0 months (range, 0.2 to 67.9 months). We performed a transplant conditioning intensity (TCI) score for the conditioning regimen (43). The TCI score ranged from 1.5 to 5.5 (median 4.5) with a median of 4.5 (range, 2.5–5.5) in the MAC group and 3.0 (range, 1.5–4.0) in the RIC group (*p*=0.000).

**Table 1:** Patient and donor characteristics.

Characteristics	Entire cohort (N=260)	Myeloid malignancies cohort (N=162)	Lymphoid malignancies cohort (N=92)	*P* values
Recipient median age, years (range)	41 (18–71)	46 (18-71)	34.5 (18-61)	0.000
Recipient sex, n (%)				0.753
Male	164 (63.1%)	101 (62.3%)	63 (64.3%)	
Female	96 (36.9%)	61 (37.7%)	35 (35.7%)	
Conditioning regimen, n (%)				0.000
MAC	203 (78.1%)	112 (69.1%)	91 (92.9%)	
RIC	57 (21.9%)	50 (30.9%)	7 (7.1%)	
HCT-CI, n (%)				0.074
0-1	236 (90.8%)	143 (88.3%)	93 (94.9%)	
≥2	24 (9.2%)	19 (11.7%)	5 (5.1%)	
Pretransplant remission status				0.000
CR	178 (68.5%)	94 (58.0%)	84 (85.7%)	
NR	82 (31.5%)	68 (42.0%)	14 (14.3%)	
ECOG, n (%)				0.007
0-1	244 (93.8%)	147 (90.7%)	97 (99.0%)	
≥2	16 (6.2%)	15 (9.3%)	1 (1.0%)	
Donor median age, years (range)	32.5 (8-64)	33 (8-64)	32 (15-60)	0.941
Donor sex, n (%)				0.088
Male	180 (69.2%)	106 (65.4%)	74 (75.5%)	
Female	80 (30.8%)	56 (34.6%)	24 (24.5%)	
Donor-recipient sex, n (%)				0.034
Female to male	51 (19.6%)	35 (21.6%)	11 (11.2%)	
Others	209 (79.2%)	127 (78.4%)	87 (88.8%)	
Blood type matching				0.588
Matched	135 (51.9%)	82 (50.6%)	53 (54.1%)	
Mismatched	125 (48.1%)	80 (49.4%)	45 (45.9%)	
PBSC graft, median (range)				
MNCs (10^8^/kg)	15.55 (3.48-41.07)	15.45 (5.2-41.07)	15.66 (3.48-32.87)	0.734
CD34^+^ cells (10^6^/kg)	11.01 (2.4-41.7)	11.00 (2.4-34.56)	11.00 (2.77-41.7)	0.995
CD3^+^ cells (10^8^/kg)	3.11 (0.44-23.32)	3.11 (0.99-9.02)	3.145 (0.44-23.32)	0.278
Umbilical cord blood, n (%)				0.000
With	84 (32.3%)	28 (17.3%)	56 (57.1%)	
Without	176 (67.7%)	134 (82.7%)	42 (42.9%)	
Umbilical cord blood cells, median (range)				
Nucleated cells (10^7^/kg)	2.28 (1.045-5.85)	2.3(1.4-4.14)	2.28 (1.045-5.85)	0.361
CD34+ cells(10^4^/kg)	6.85 (0.36-14)	6.91 (0.36-12.7)	6.85 (0.91-14)	0.016
Median follow-up months, (range)	27.0 (0.2-67.9)	28.0 (0.2-67.0)	26.0 (0.9-67.9)	0.593

N, number of patients; MAC, myeloablative conditioning; RIC, Reduced-intensity conditioning; HCT-CI, Hematopoietic Cell Transplantation Comorbidity Index; CR, complete response; NR, non-remission; ECOG, Eastern Cooperative Oncology Group score standard; PBSC, peripheral blood stem cell; MNC, mononuclear cell.

### 3.2 Graft failure

Graft failure was observed in nine patients (3.46%), of which only one (1.02%) occurred in lymphoid malignancies and the remaining eight (4.94%) developed in myeloid malignancies (*p*=0.050). PGF accounted for six (2.31%) and SGF for three (1.15%). Five out of the nine patients received a second transplant, of which two patients achieved long-term survival. The median time for neutrophil engraftment was 12 days (range, 9–28 days), while the median time for platelet engraftment was 13 days (range, 9–87 days).

### 3.3 GVHD

**Figure 1:**
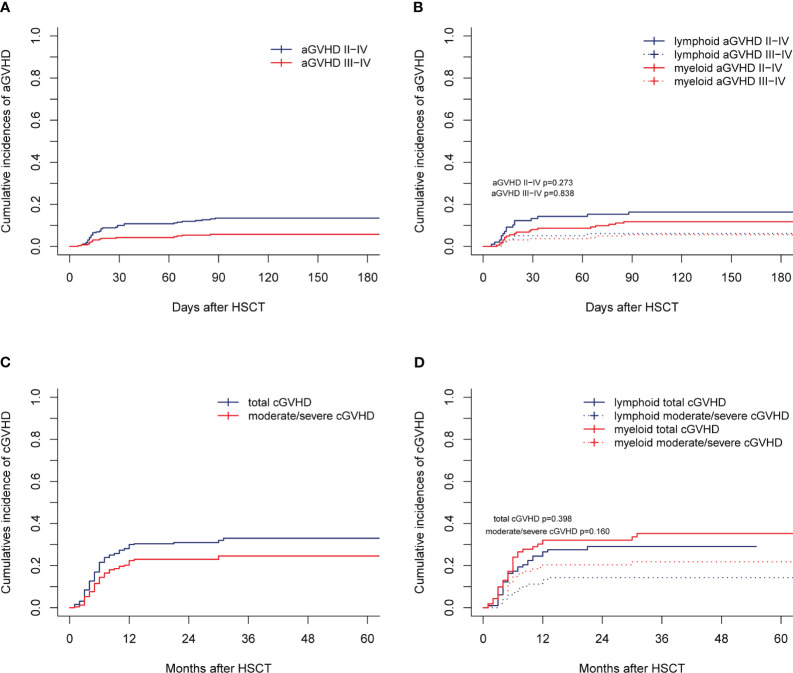
aGVHD and cGVHD of the entire cohort. Cumulative incidences are shown for **(A, B)** aGVHD and **(C, D)** cGVHD.

For the entire cohort, the CIs of grade I-IV, II-IV, and III-IV aGVHD by 28 days were 26.15% (95% confidence interval [CI], 20.97%-31.64%), 9.23% (95% CI, 6.10%-13.14%), and 4.23% (95% CI, 2.24%-7.18%), respectively. The 180-day CIs of grade I-IV, II-IV, and III-IV aGVHD were 35.00% (95% CI, 29.24%-40.81%), 13.46% (95% CI, 9.64%-17.92%), and 5.77% (95% CI, 3.37%-9.07%), respectively (**Figure 1A**). The CIs of grade II-IV (*p*=0.273) and III-IV aGVHD (*p*=0.838) were similar between the myeloid and lymphoid malignancies (**Figure 1B**), although the CI of grade I-IV aGVHD in myeloid malignancies was significantly lower than that in lymphoid malignancies [29.01% (95% CI, 22.22%-36.14%) *vs* 41.84% (95% CI, 31.94%-51.41%), *p*=0.003)]. The CIs of total and moderate/severe cGVHD in all patients within 2 years after transplantation were 30.97% (95% CI, 25.43%-36.66%) and 18.08% (95% CI, 13.68%-22.98%), respectively (**Figure 1C**). The CIs of total and moderate/severe cGVHD between myeloid and lymphoid malignancies were similar (*p*=0.398 and *p*=0.160, respectively) (**Figure 1D**). In our long-term follow-up, the proportion of patients with cGVHD requiring second-line therapy was 28.05% (23/82).

### 3.4 Survival outcomes for the entire cohort

**Figure 2:**
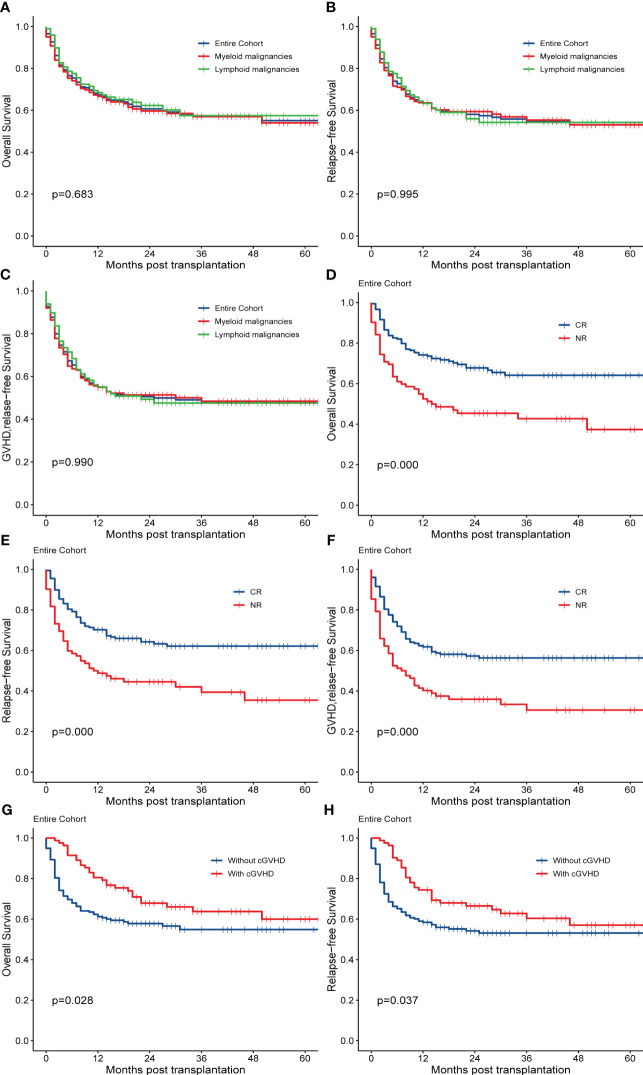
Survival outcomes of the entire cohort. OS, RFS, and GRFS are shown for **(A–C)** by disease type, and **(D–F)** by disease status at transplantation. OS and RFS are shown for **(G, H)** by the status of cGVHD.

By the end of the follow-up, 104 patients died. Relapse (n=43) and infection (n=34) accounted for 74.04% of the death. The 2-year OS, RFS, and GRFS for the entire cohort were 60.70% (95% CI, 54.80%-67.10%), 58.10% (95% CI, 52.20%-64.50%), and 50.60% (95% CI, 44.80%-57.10%), respectively. There were no significant differences in OS (*p=*0.683, **Figure 2A**), RFS (*p=*0.995, **Figure 2B**), and GRFS (*p=*0.990, **Figure 2C**) between myeloid and lymphoid malignancies. All results of the univariate analyses are included in the **Supplementary Material**. In the multivariate Cox analysis (**Table 2**), disease status at transplantation was an independent prognostic factor for OS (HR,1.9; 95% CI,1.3-2.9; *p*=0.002), RFS (HR, 1.8; 95% CI, 1.2-2.7; *p*=0.005), and GRFS (HR, 1.7; 95% CI, 1.2-2.5; *p*=0.004). The patients with CR at transplantation had significantly higher 2-year OS (67.70% *vs* 45.40%, *p*=0.000, **Figure 2D**), RFS (64.30% *vs* 44.50%, *p*=0.000, **Figure 2E**), and GRFS (57.30% *vs* 36.00%, *p*=0.000, **Figure 2F**) than those of patients with NR. Meanwhile, patients without cGVHD had significantly lower OS (HR, 0.55; 95% CI, 0.35-0.87; *p*=0.010) and RFS (HR, 0.57; 95% CI, 0.37-0.87; *p*=0.009) than those with cGVHD. The 2-year OS and RFS for patients without cGVHD were 57.83% and 54.21%, respectively, while for patients with cGVHD, these were 67.85% and 66.50%, respectively (*p*=0.028, **Figure 2G** for OS; *p*=0.037, **Figure 2H** for RFS). ECOG scores also had a strong trend toward lowering the OS (HR, 1.9; 95% CI, 0.97-3.7; *p*=0.060), RFS (HR, 1.9; 95% CI, 0.99-3.8; *p*=0.054), and GRFS (HR, 1.7; 95% CI, 0.92-3.1; *p*=0.089).

**Table 2:** Multivariate analysis for OS, RFS, GRFS, NRM, and relapse of the entire cohort.

OS	p. value	HR (95% CI for HR)
Recipient age (>median age vs ≤median age)	0.260	1.3 (0.83-2)
Disease status (NR *vs* CR)	0.002	1.9 (1.3-2.9)
Regimen (RIC *vs* MAC)	0.460	1.2 (0.73-2)
ECOG (2-4 *vs* 0-1)	0.060	1.9 (0.97-3.7)
cGVHD (with *vs* without)	0.010	0.55 (0.35-0.87)
RFS		
Recipient sex (female *vs* male)	0.260	0.79 (0.52-1.2)
Recipient age (>median age vs ≤median age)	0.350	1.2 (0.8-1.9)
Disease status (NR *vs* CR)	0.005	1.8 (1.2-2.7)
Regimen (RIC *vs* MAC)	0.670	1.1 (0.68-1.8)
ECOG (2-4 *vs* 0-1)	0.054	1.9 (0.99-3.8)
cGVHD (with *vs* without)	0.009	0.57 (0.37-0.87)
GRFS		
Recipient age (>median age vs ≤median age)	0.380	1.2 (0.81-1.8)
Disease status (NR *vs* CR)	0.004	1.7 (1.2-2.5)
Regimen (RIC *vs* MAC)	0.680	1.1 (0.7-1.7)
ECOG (2-4 *vs* 0-1)	0.089	1.7 (0.92-3.1)
NRM		
Recipient age (>median age vs ≤median age)	0.006	2.24 (1.266-3.96)
Disease status (NR *vs* CR)	0.470	1.21 (0.714-2.07)
Regimen (RIC *vs* MAC)	0.270	1.36 (0.783-2.38)
ECOG (2-4 *vs* 0-1)	0.140	1.73 (0.840-3.55)
aGVHD (grade II-IV *vs* grade 0-I)	0.049	1.81 (1.003-3.26)
Relapse		
Disease status (NR *vs* CR)	0.021	1.909 (1.104-3.30)
cGVHD (with *vs* without)	0.052	0.529 (0.279-1.01)

OS, overall survival; RFS, relapse-free survival; GRFS, graft-versus-host disease-free, relapse-free survival; NRM, non-relapse mortality; NR, non-remission; CR, complete response; RIC, Reduced-intensity conditioning; MAC, myeloablative conditioning; ECOG, Eastern Cooperative Oncology Group score standard; cGVHD, chronic graft-versus-host disease; PBSC, peripheral blood stem cell.

### 3.5 Survival outcomes for myeloid malignancies

The 2-year OS, RFS, and GRFS for myeloid malignancies were 59.70% (95% CI, 52.40% - 68.10%), 59.40% (95% CI, 52.20% - 67.50%), and 51.40% (95% CI, 44.10% - 59.80%), respectively. In multivariate analysis, disease status at transplantation was the independent prognostic factor for OS (HR, 1.9; 95% CI, 1.1-3.2; *p*=0.018), RFS (HR, 1.8; 95% CI, 1.1-3.0; *p*=0.021), and GRFS (HR, 1.7; 95% CI, 1.1-2.7; *p*=0.029) (**Table 3**). Patients with CR at transplantation had significantly higher 2-year OS (69.50% *vs* 46.20%, *p*=0.001, **Figure S2A**), RFS (68.90% *vs* 46.20%, *p*=0.001, **Figure S2B**), and GRFS (61.50% *vs* 37.30%, *p*=0.001, **Figure S2C**) than those with NR. Higher ECOG scores (2–4) were associated with inferior survival outcomes in OS (HR, 2.4; 95% CI, 1.2-4.8; *p*=0.014), RFS (HR, 2.4; 95% CI, 1.2-4.8; *p*=0.014), and GRFS (HR, 1.9; 95% CI, 1.7-2.7; *p*=0.050) (**Table 3**). The 2-year OS, RFS, and GRFS for patients with ECOG scores of 2-4 were 33.30%, 33.30%, and 26.70%, respectively, while for patients with ECOG scores of 0-1, these were 62.50%, 62.20%, and 54.00%, respectively (*p*=0.005, **Figure S2D** for OS; *p*=0.004, **Figure S2E** for RFS; and *p*=0.010, **Figure S2F** for GRFS, respectively). cGVHD was associated with better outcomes for 2-year OS (72.50% *vs* 53.40%; *p*=0.019, **Figure S2G**) and 2-year RFS (71.90% *vs* 53.00%; *p*=0.038, **Figure S2H**).

**Table 3:** Multivariate analysis for OS, RFS, GRFS, NRM, and relapse of myeloid malignancies.

OS	p. value	HR (95% CI for HR)
Recipient sex (female *vs* male)	0.140	0.66 (0.38-1.1)
Recipient age (>median age vs ≤median age)	0.220	1.4 (0.83-2.2)
Disease status (NR *vs* CR)	0.018	1.9 (1.1-3.2)
ECOG (2-4 *vs* 0-1)	0.014	2.4 (1.2-4.8)
cGVHD (with *vs* without)	0.004	0.42 (0.24-0.76)
RFS		
Recipient sex (female *vs* male)	0.200	0.7 (0.41-1.2)
Recipient age (>median age vs ≤median age)	0.260	1.3 (0.81-2.1)
Disease status (NR *vs* CR)	0.021	1.8 (1.1-3.0)
ECOG (2-4 *vs* 0-1)	0.014	2.4 (1.2-4.8)
aGVHD (grade I-IV *vs* grade 0)	0.310	0.74 (0.42-1.3)
cGVHD (with *vs* without)	0.021	0.50 (0.28-0.9)
GRFS		
Recipient age (>median age vs ≤median age)	0.150	1.4 (0.88-2.2)
Disease status (NR *vs* CR)	0.029	1.7 (1.1-2.7)
ECOG (2-4 *vs* 0-1)	0.050	1.9 (1-3.6)
Donor–recipient blood type (mismatched *vs* matched)	0.260	1.3 (0.82-2)
PBSC graft MNCs	0.160	0.97 (0.94-1)
NRM		
Recipient sex (female *vs* male)	0.250	0.657 (0.322-1.34)
Recipient age (>median age vs ≤median age)	0.028	2.319 (1.093-4.92)
Disease status (NR *vs* CR)	0.310	1.427 (0.717-2.84)
Regimen (RIC *vs* MAC)	0.550	1.233 (0.620-2.45)
ECOG (2-4 *vs* 0-1)	0.250	1.599 (0.724-3.53)
PBSC graft CD34^+^cells	0.097	0.947 (0.889-1.01)
Relapse		
Disease status (NR *vs* CR)	0.066	1.95 (0.957-3.97)

OS, overall survival; RFS, relapse-free survival; GRFS, graft-versus-host disease-free, relapse-free survival; NRM, non-relapse mortality; NR, non-remission; CR, complete response; RIC, Reduced-intensity conditioning; MAC, myeloablative conditioning; ECOG, Eastern Cooperative Oncology Group score standard; cGVHD, chronic graft-versus-host disease; aGVHD, acute graft-versus-host disease; PBSC, peripheral blood stem cell; MNC, mononuclear cell.

### 3.6 Survival outcomes for lymphoid malignancies

The 2-year OS, RFS, and GRFS were 62.30% (95% CI, 53.10%-73.00%), 56.0% (95% CI, 46.70%- 67.10%), and 49.3% (95% CI, 40.20% - 60.50%), respectively. In multivariate analysis, disease status at transplantation was the independent prognostic factor for OS (HR, 2.6; 95% CI, 1.1-5.8; *p*=0.023), RFS (HR, 2.4; 95% CI, 1.1-5.2; *p*=0.029), and GRFS (HR, 2.1; 95% CI, 1.0-4.4; *p*=0.037) (**Table 4**). Patients with CR at transplantation had significantly higher 2-year OS (65.80% *vs* 42.90%, *p*=0.095, **Figure S3A**), RFS (59.1% *vs* 35.7%, *p*=0.053, **Figure S3B**), and GRFS (52.70% *vs* 28.60%, *p*=0.037, **Figure S3C**) than those with NR. RIC regimen was also associated with inferior survival outcomes in OS (HR, 5.3; 95% CI, 2.0-14.0; *p*=0.001), RFS (HR, 4.8; 95% CI, 1.8-13.0; *p*=0.002), and GRFS (HR, 4.6; 95% CI, 1.9-11.0; *p*=0.001). The 2-year OS, RFS, and GRFS for patients with the RIC regimen were 28.57%, 28.57%, and 28.60%, respectively, while for patients with the MAC regimen, these were 64.90%, 58.20%, and 52.70%, respectively (*p*=0.005, **Figure S3D** for OS, *p*=0.010, **Figure S3E** for RFS and *p*=0.001, **Figure S3F** for GRFS, respectively). Higher HCT-CI scores (≥2) were associated with worse outcomes for 2-year OS (20.00% *vs* 64.50%; *p*=0.019, **Figure S3G**) and 2-year RFS (20.00% *vs* 57.90%; *p*=0.040, **Figure S3H**).

**Table 4:** Multivariate analysis for OS, RFS, GRFS, NRM, and relapse of lymphoid malignancies.

OS	p. value	HR (95% CI for HR)
Disease status (NR *vs* CR)	0.023	2.6 (1.1-5.8)
Regimen (RIC *vs* MAC)	0.001	5.3 (2.0-14)
HCT-CI (≥2 *vs* 0-1)	0.004	4.9 (1.6-14)
RFS		
Disease status (NR *vs* CR)	0.029	2.4 (1.1-5.2)
Regimen (RIC *vs* MAC)	0.002	4.8 (1.8-13.0)
HCT-CI (≥2 *vs* 0-1)	0.015	3.8 (1.3-11.0)
aGVHD (grade II-IV *vs* grade 0-I)	0.140	1.7 (0.83-3.6)
GRFS		
Disease status (NR *vs* CR)	0.037	2.1 (1.0-4.4)
Regimen (RIC *vs* MAC)	0.001	4.6 (1.9-11.0)
Donor-recipient sex (female-male *vs* others)	0.210	0.55 (0.21-1.4)
NRM		
HCT-CI (≥2 *vs* 0-1)	0.007	3.77 (1.427-9.95)
aGVHD (grade I-IV *vs* grade 0)	0.250	1.79 (0.664-4.82)
aGVHD (grade II-IV *vs* grade 0-I)	0.190	1.92 (0.731-5.05)
Relapse		
Recipient age (>median age vs ≤median age)	0.110	0.386 (0.121-1.23)
Disease status (NR *vs* CR)	0.052	2.662 (0.990-7.16)
Donor age	0.810	1.005 (0.962-1.05)

OS, overall survival; RFS, relapse-free survival; GRFS, graft-versus-host disease -free, relapse-free survival; NRM, non-relapse mortality; NR, non remission; CR, complete response; RIC, Reduced-intensity conditioning; MAC, myeloablative conditioning; HCT-CI, Hematopoietic Cell Transplantation Comorbidity Index; ECOG, Eastern Cooperative Oncology Group score standard; aGVHD, acute graft-versus-host disease.

### 3.7 NRM

**Figure 3:**
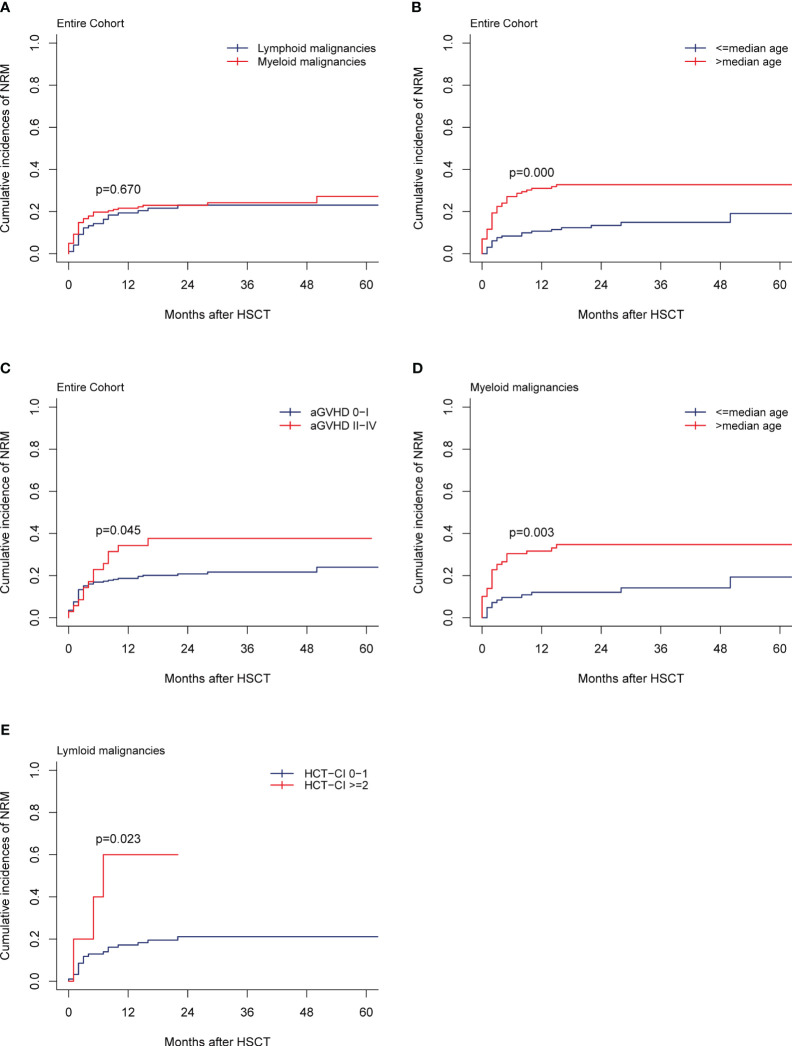
NRM. **(A)** NRM by disease type of the entire cohort, **(B, C)** NRM by the median recipient age, the grade of aGVHD, **(D)** NRM by the median recipient age of the myeloid malignancies, **(E)** NRM by the HCT-CI scores of the lymphoid malignancies.

For the entire cohort, the 1-year and 2-year NRMs were 20.77% (95% CI,16.07%-25.90%) and 23.04% (95% CI, 18.06%-28.40%), respectively. In total, 61 patients died from NRM, which accounted for 58.65% (61/104) of all deaths. Infection was the most common cause of NRM, accounting for 42.62% (26/61), followed by cGVHD at 19.67% (12/61), organ failure at 18.03% (11/61), hemorrhagic diseases at 8.20% (5/61), graft failure at 6.55% (4/61), and aGVHD at 4.92% (3/61). The 2-year NRMs between myeloid and lymphoid malignancies were similar (22.99% *vs* 23.08%, *p=*0.670; **Figure 3A**). In multivariate analysis, older patients (>median age) (HR, 2.42; 95% CI, 1.266-3.96; *p*=0.006) and grade II-IV aGVHD (HR, 1.81; 95% CI, 1.003-3.26; *p*=0.049) were the unfavorable independent prognostic factors for NRM (**Table 2**). The 2-year NRMs were significantly increased in older patients (32.76% *vs* 13.43%; *p=*0.000, **Figure 3B**) and patients with grade II-IV aGVHD (37.65% *vs* 20.78%; p=0.045, **Figure 3C**). For myeloid malignancies, the multivariate analysis results showed that older patients had a significantly increased NRM compared with younger patients (12.05% *vs* 34.75%; *p=*0.003, **Figure 3D**) (**Table 3**). For lymphoid malignancies, higher HCT-CI scores (≥2) significantly increased the NRM (50.00% *vs* 15.84%; *p=*0.023, **Figure 3E**) (**Table 4**).

### 3.8 Relapse

**Figure 4:**
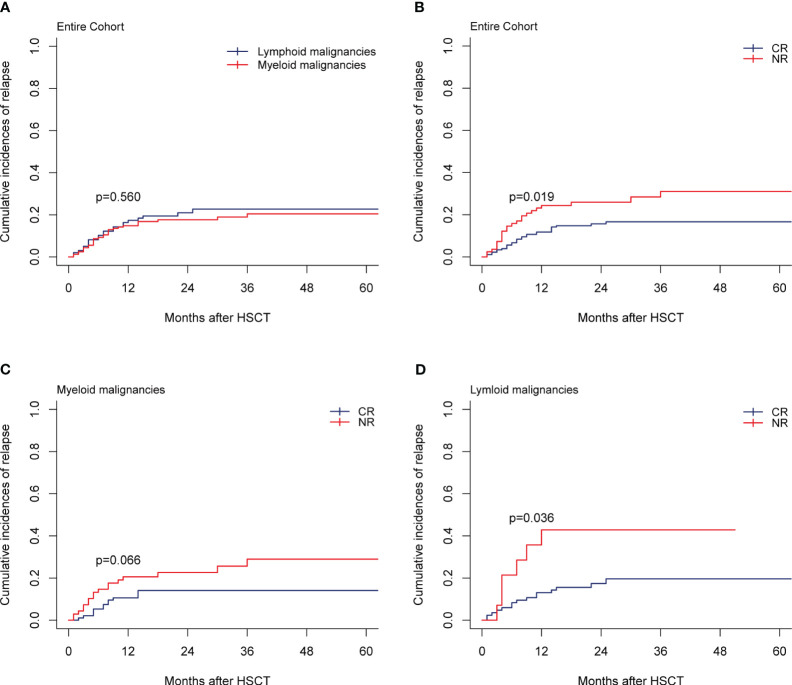
Relapse. **(A)** Relapse by disease type of the entire cohort, **(B–D)** Relapse by disease status at transplantation of the entire cohort, myeloid malignancies, and lymphoid malignancies.

In total, 43 patients relapsed in the entire cohort and the median time of relapse was post-transplant 16 months (range, 1-36 months). For the entire cohort, the 1-year and 2-year CIs of relapse were 15.77% (95% CI, 11.64%-20.46%) and 18.90% (95% CI, 14.33%-23.97%), respectively. CIs of relapse were similar between myeloid and lymphoid malignancies (*p=*0.560; **Figure 4A**). In multivariate analysis for the entire cohort, the disease status was the independent prognostic factor for relapse, while cGVHD had a strong trend toward lowering the relapse rate (**Table 2**). CR status at transplantation significantly lowered the 2-year CI of relapse as compared with NR status (15.66% *vs* 25.98%; *p*=0.019, **Figure 4B**). NR status at transplantation also had strong trends of increasing the risk of relapse for myeloid (HR, 1.95; 95% CI, 0.957-3.97; *p*=0.066) and lymphoid malignancies (HR, 2.662; 95% CI, 0.990-7.16; *p*=0.052). The 2-year CI of relapse of patients with NR status at transplantation was higher than that with CR status, whether for myeloid (22.60% *vs* 14.08%; *p*=0.066, **Figure 4C**) or for lymphoid malignancies (42.86% *vs* 17.41%; *p*=0.036, **Figure 4D**). The rate of HLA loss in relapsed patients was 14.28% (2/14). We carried out maintenance treatments for patients with tyrosine kinase inhibitors such as sorafenib for FMS-like tyrosine kinase 3 internal tandem duplication (FLT3-ITD) mutated AML and dasatinib for Philadelphia chromosome-positive ALL (Ph^+^ ALL). Preemptive treatment was adopted for patients with measurable residual disease (MRD, flow cytometry, and/or reverse transcription-polymerase chain reaction) relapse. Azacytidine in combination with interferon α was given to 49 patients with MRD-positive myeloid malignancies including AML and MDS, of which 20/24 (83.33%) achieved MRD-negative after 6 cycles of therapy.

### 3.9 Infection and other complications

The median time of CMV and EBV reactivation was 114 days (range,15-1041) and 429 days (range, 41 - 939) after transplantation, respectively. The 1-year CIs of CMV and EBV reactivation were 43.46% (95% CI, 37.39%-49.37%) and 18.08% (95% CI, 13.68%-22.98%) in the entire cohort. The 1-year CIs of CMV and EBV reactivation were similar between myeloid and lymphoid malignancies (*p=*0.147, *p=*0.482). In the entire cohort, 28 patients (10.77%) had CMV disease, and 7 (2.69%) developed post-transplant lymphoproliferative disorder (PTLD), of which 5 were associated with EBV reactivation. No statistically significant differences were found between the 2-year CIs of PTLD between myeloid and lymphoid malignancies [2.47% (95% CI, 0.81%-5.80%) vs 3.06% (95% CI, 0.82-7.98%), *p*=0.764]. The CI of BKV-related hemorrhagic cystitis was 16.54%, which had no difference between myeloid and lymphoid malignancies (*p*=0.600). A total of 10 patients showed activation of human herpesvirus-6 B (HHV-6B) after transplantation. All patients were included in immune reconstitution studies and 81 cases were analyzed at each endpoint. On days +120, median CD3^+^, CD4^+^, CD8^+^, CD19^+^, and CD56/CD16^+^ counts were 954 (95-4891), 180 (8-743),754 (73-4272), 34 (1-276), and 215 (19-2539)/μl, respectively.

## 4 Discussion

Data from the large sample, long-term follow-up retrospective study support that the low-dose ATG/PTCy-based regimen has a powerful efficacy in the prevention of aGVHD for patients who underwent haplo-PBSCT. The regimen also brings out a relatively lower reactivation incidence of CMV and EBV. The CI of grade II-IV aGVHD was only 13.46% (95% CI, 9.64%-17.92%) in this study, which was lower than 20%-42.4% for the ATG-based regimen (44–50) or 22%-59% for the PTCy-based regimen (21, 51–55), while the CI of total cGVHD (30.97%) was close to that of the ATG-based (17%-41.4%) or PTCy-based (21%-41%) regimens. The main studies evaluating CIs of GVHD in patients with hematologic malignancies receiving haplo-HSCT based on different dose ATG regiments are shown in **Table 5**. A large cohort study (n=441) from the Acute Leukemia Working Party of the European Society for Blood and Marrow Transplantation (EBMT) showed that the addition of ATG (2.5-10 mg/kg) to standard-dose PTCy is feasible and achieved similar transplantation outcomes, especially for a lower incidence of cGVHD as comparable with PTCy alone (53). More recently, the varying combinations of ATG (2-10 mg/kg) and PTCy (80-100 mg/kg) for GVHD prophylaxis have been reported and the results showed that the combination regimens could lower the incidences of GVHD (25–28, 56–60) with the CIs for grade II-IV aGVHD and total cGVHD ranging from 11.1% to 34.6% and 17.0% to 36.5%, respectively. The incidence of grade II-IV aGVHD in our study was similar, even lower than that of other combination regimens, although the doses of ATG and PTCy in our regimen were lower than those in large doses of ATG-based or standard dose of PTCy-based combination regimens. Not only was the CI of total cGVHD comparable, but the incidence of moderate/severe cGVHD (18.08%) in the present study was also comparable to that of standard-dose PTCy plus lower-dose ATG-based (13.5-20.2%) (27, 56, 60, 61) or large-dose ATG plus lower-dose PTCy-based (15.4%-17%) regimens (25, 62). These results indicated that the large dose of ATG or standard dose of PTCy was not indispensable in their combination. ATG affects the immune system in a variety of ways, including TCD in peripheral lymphoid tissues and the blood, modulation of key cell surface molecules that mediate leukocyte/endothelium interactions, induction of B lineage cell apoptosis, interference with the function of other immune effectors like dendritic cells, interactions with dendritic cell functional properties, and induction of regulatory T cells (Tregs) and natural killer (NK) cells (63). Due to the poor expression of aldehyde dehydrogenase 1A1 (ALDH1A1) in alloreactive T cells, previous studies have revealed that PTCy may effectively eliminate these cells (64, 65). However, recent research has shown that the mechanism of PTCy for GVHD prophylaxis is more intricate than previously believed. In a mouse model, it was found that PTCy inhibited the proliferation of alloreactive CD4^+^ T cells, reconstituted CD4^+^ Tregs preferentially, and caused functional impairment in both alloreactive CD4^+^ and CD8^+^ T cells. Severe GVHD occurred when Foxp3+ Tregs were selectively depleted, and the importance of Tregs in GVHD prophylaxis after PTCy has been observed (66). Recent studies have also highlighted that PTCy promotes myeloid suppressor cell proliferation, which is the important mediator of T cell function (67). Their different mechanisms of ATG and PTCy for the prevention of GVHD might be the major reason for their combination strengthening the efficacy of GVHD prophylaxis. Only a 1-day dose of PTCy was used in the present study, which might be the major reason for the relatively high incidence of cGVHD because only a 1-day dose of PTCy (50 mg) could result in a higher incidence of extensive cGVHD as compared with a standard 2-day dose of PTCy (100 mg) for haplo-HSCT (7). More patients with active disease at transplantation and more female donors in the current study may also be attributable to a relatively high incidence of cGVHD (16, 68).

**Table 5:** Main studies evaluating ATG-based protocol in haplo-HSCT.

Reference	N	Conditioning regimen	ATG (mg/kg)	II-IV aGVHD	III-IV aGVHD	Total cGVHD	moderate-severe cGVHD	Survival
Peccatori, 2015 (44)	121	Treosulfan/Flu	10	35 ± 9%	22 ± 8%	47 ± 11%	NA	3-y OS 25%
Luo, 2014 (45)	99	Bu/Cy	10	42.4%	17.2%	41.4%	NA	5-y OS 60.8%
Long, 2016 (46)	105	Bu/Cy/Flu	12.5	21.9 ± 7.8%	14.3 ± 6.7%	24.1 ± 9.4	NA	3-y OS 52.6 ± 10.4%
Lee, 2011 (47)	83	Bu/Flu	12	20%	7%	34%	24%	3-y EFS 60%
Ikegame, 2015 (48)	34	Bu/Flu	8	30.7%	NA	NA	20%	1-y OS 42.3%/62.5%
Huang, 2016 (49)	130	Bu/Cy	10	33.4%	14.9%	38.6%	16.5%	3-y OS 45.6% ± 5.6%
Di Bartolomeo, 2013 (50)	80	Bu/Flu/thiotepa	20	24 ± 0.2%	5 ± 0.6%	17 ± 0.3%	6 ± 0.1%	3-y OS 45%

N, number of patients; aGVHD, acute graft-versus-host disease; cGVHD, chronic graft-versus-host disease; Flu, fludarabine; ATG, anti-thymocyte globulin; Bu, busulfan; Cy, cyclophosphamide; OS, overall survival; NA, not available; EFS, event-free survival.

Relatively better survival outcomes were achieved in the present study with the 2-year OS of 60.70% and RFS of 58.10%, although nearly one-third of patients were in active disease status at transplantation. In the multivariate Cox analysis, the disease status had significant adverse effects on OS, RFS, and GRFS in the entire cohort as well as in myeloid and lymphoid malignancies (69). cGVHD as a favorable prognostic factor was associated with superior OS and RFS for the entire cohort and myeloid malignancies, but not for lymphocyte malignancies. Bhatt’s recent study showed an overall more favorable effect of cGVHD for patients with AML and MDS, which suggested that adult patients who developed cGVHD achieved a longer OS compared with those without cGVHD (70). Although the outcomes of patients with active disease or without cGVHD are discouraging, they are similar to those with other transplantation approaches (69, 70). HCT-CI affected the survival of OS and RFS for patients with lymphoid malignancies but did not for all patients and patients with myeloid malignancies. A total of four out of five patients with high HCT-CI (≥2) scores died of lymphocyte malignancies, which may be a false positive result due to selection bias. ECOG scores negatively affected the survival of all patients and patients with myeloid. We did not include ECOG scores in the analysis for lymphoid malignancies due to the small number of patients (1.02%, 1/98) with higher ECOG scores (≥2). RIC was associated with an inferior survival outcome for lymphoid malignancies in our study, which was consistent with previous findings (71).

Mortality from infection and GVHD accounted for the vast majority of NRM in haplo-HSCT. In the multivariate analysis, older patients and grade II-IV aGVHD were the unfavorable independent factors for the entire cohort, whereas only age was associated with the NRM of patients with myeloid malignancies and HCT-CI was associated with the NRM of patients with lymphoid malignancies. Grade II-IV aGVHD only affected the NRM of patients in the entire cohort, but did not for patients with myeloid and lymphoid malignancies, which might be related to there being only 19 (11.73%) in myeloid malignancies and 16 (16.33%) in lymphoid malignancies with grade II-IV aGVHD. The 2-year CI of NRM in our study was 23.04%, which was similar to the results in haplo-HSCT with PTCy-based (21, 53) and ATG-based GVHD prophylaxis regimens (14.8-34.9%) (45, 46, 49). The relatively high NRM may be related to there being more older patients with a median age of 41 years old in this study. The 2-year CI of relapse was 18.90% in the present study, which was similar to that of 14.2%-21% from other studies (21, 45, 46, 50, 72), although nearly one-third of patients were in active disease status at transplantation. The relapse rate is comparable to that in Ruggeri’s (21) and Salvatore’s studies (72), both of which were with 100% CR patients. These results indicated that the low-dose ATG/PTCy-based regimen did not increase the risk of relapse. In the multivariate analysis, disease status at transplantation as an independent prognostic factor significantly affected the risk of relapse for all patients, patients with lymphoid and myeloid malignancies, while cGVHD only affected the risk of relapse for all patients, but did not for patients with lymphoid and myeloid malignancies. This might be related to a lower number of patients with lymphoid (28.57%) and myeloid (33.33%) malignancies developing cGVHD.

The 1-year CIs of CMV and EBV reactivation were 43.46% and 18.08% for all patients in the present study, respectively. In terms of incidences of viral reactivations, studies have had different results for the combination of ATG and PTCy (25, 26, 51–54, 58). In our study, the CMV reactivation rate was significantly lower than that of 49.5%-64% for the large dose ATG-based regimen (6, 46, 49) and similar to that of 38%-50% for the standard PTCy-based regimen (7, 23, 73). The EBV reactivation rate was also lower than that of 40%-50.5% for ATG-based regimens (46, 49), while it was similar to that of 8%-11.9% for PTCy-based regimens (51, 55, 73). The pivotal reason for lower incidences of virus reactivation is the relatively faster recovery of CD4^+^ T cells with PTCy (17) and the low-dose ATG/PTCy-based (29) regimens. Tischer et al. retrospectively compared the incidences of viral infection between ATG-based and PTCy-based regimens, and the results showed that both CMV reactivation rate and virus infection-related mortality (VIRM) in the PTCy group were lower than those in the ATG group (CMV reactivation rates: 31% *vs* 57%; 1-year VIRM, 0% *vs* 29%; *p*=0.009) *(* 17). As well described by a Center for International Blood and Marrow Transplant Research (CIBMTR) study, PTCy is associated with a higher incidence of CMV infection and can abrogate the benefit of a lower incidence of cGVHD (74). CMV reactivation did show an adverse effect on moderate/severe cGVHD in our study (HR, 2.93; 95% CI,1.59-5.39; *p*=0.001). However, recent studies have shown that the availability of letermovir as prophylaxis in the first 100 days after transplantation is expected to positively contribute to the outcomes and may restore its original benefit on cGVHD (75, 76). In our study, no patients received letermovir as prophylaxis, which may explain our failure to reduce the rate of viral activation.

According to the results from the large sample retrospective study with a long-term follow-up, it was demonstrated that the low-dose ATG/PTCy-based regimen has an outstanding efficacy for preventing the occurrence of aGVHD after haplo-PBSCT without increasing the risk of disease relapse. The study also has some limitations, although the study has a large sample size with a long-term follow-up. First, this is a single-center, retrospective study. Second, the study included relatively complex characteristics of patients and donors, such as various kinds of hematologic malignancies and the range of age from 18 to 71. These might bring out the difficulty of the result analysis. Third, the sample size of lymphoid malignancies should be increased because it was lower than 100 cases in the present study. Additional well-designed trials with sizable populations of each type of donor and graft source, as well as the indicated relative precautions, ought to be carried out to address this problem.

## Data availability statement

The original contributions presented in the study are included in the article/**Supplementary Material**. Further inquiries can be directed to the corresponding authors.

## Ethics statement

The studies involving humans were approved by Ethics committee of Shanghai General Hospital. The studies were conducted in accordance with the local legislation and institutional requirements. Written informed consent for participation was not required from the participants or the participants’ legal guardians/next of kin in accordance with the national legislation and institutional requirements.

## Author contributions

This study was conceived and designed by XMS; XYL and JY analyzed and interpreted the data and wrote the manuscript; YC, CMH, XWX, HYQ, JHN, KZ, YZ, XXX, YW, CS, YT, BXD, and LPW took care of patients in clinical practice.
