# Supplementary material for: Low-dose anti-thymocyte globulin plus low-dose post-transplant cyclophosphamide-based regimen for prevention of graft-versus-host disease after haploidentical peripheral blood stem cell transplants: a large sample, long-term follow-up retrospective study
Source: Front Immunol. 2023 Oct 26;14:1252879. doi: 10.3389/fimmu.2023.1252879 (PMC10639171; doi:10.3389/fimmu.2023.1252879)
Supplement: Supplementary file 1 [file DataSheet_1.docx]

Supplementary Material

**
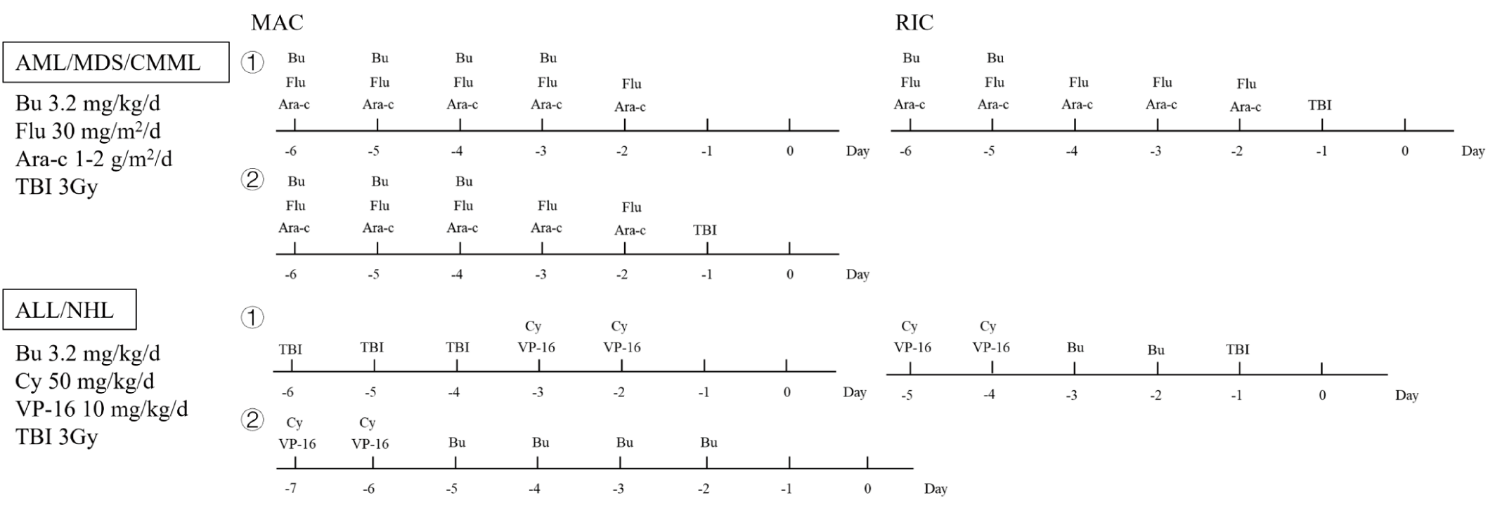
**

**Supplementary Figure 1.** The schema of conditioning regimens for myeloid and lymphoid malignancies.


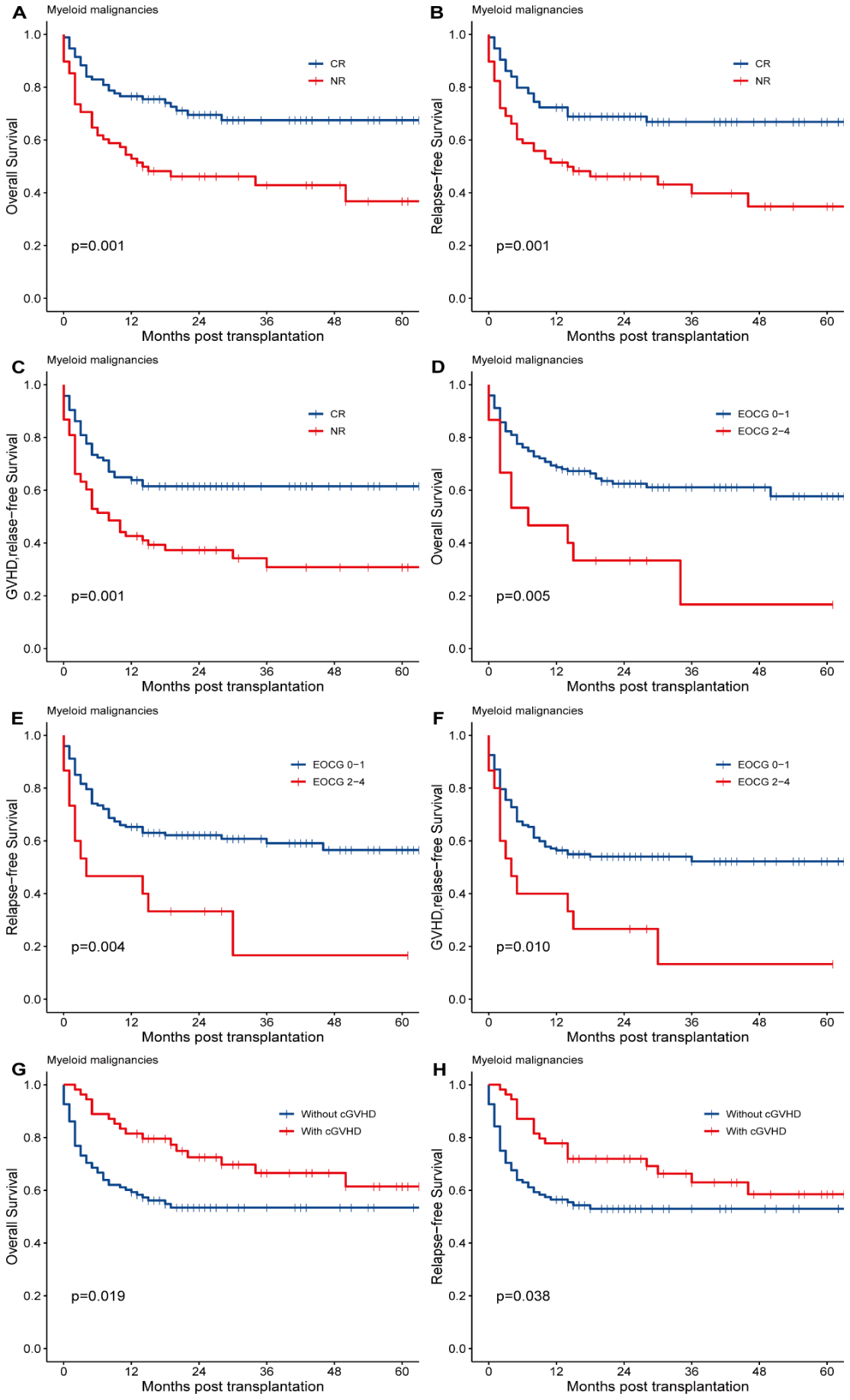


**Supplementary Figure 2.** Survival outcomes of the myeloid malignancies. OS, RFS, and GRFS are shown for A, B, and C by disease status at transplantation, and for D, E, and F by the EOCG scores. OS and RFS are shown for G, and H by the status of cGVHD.


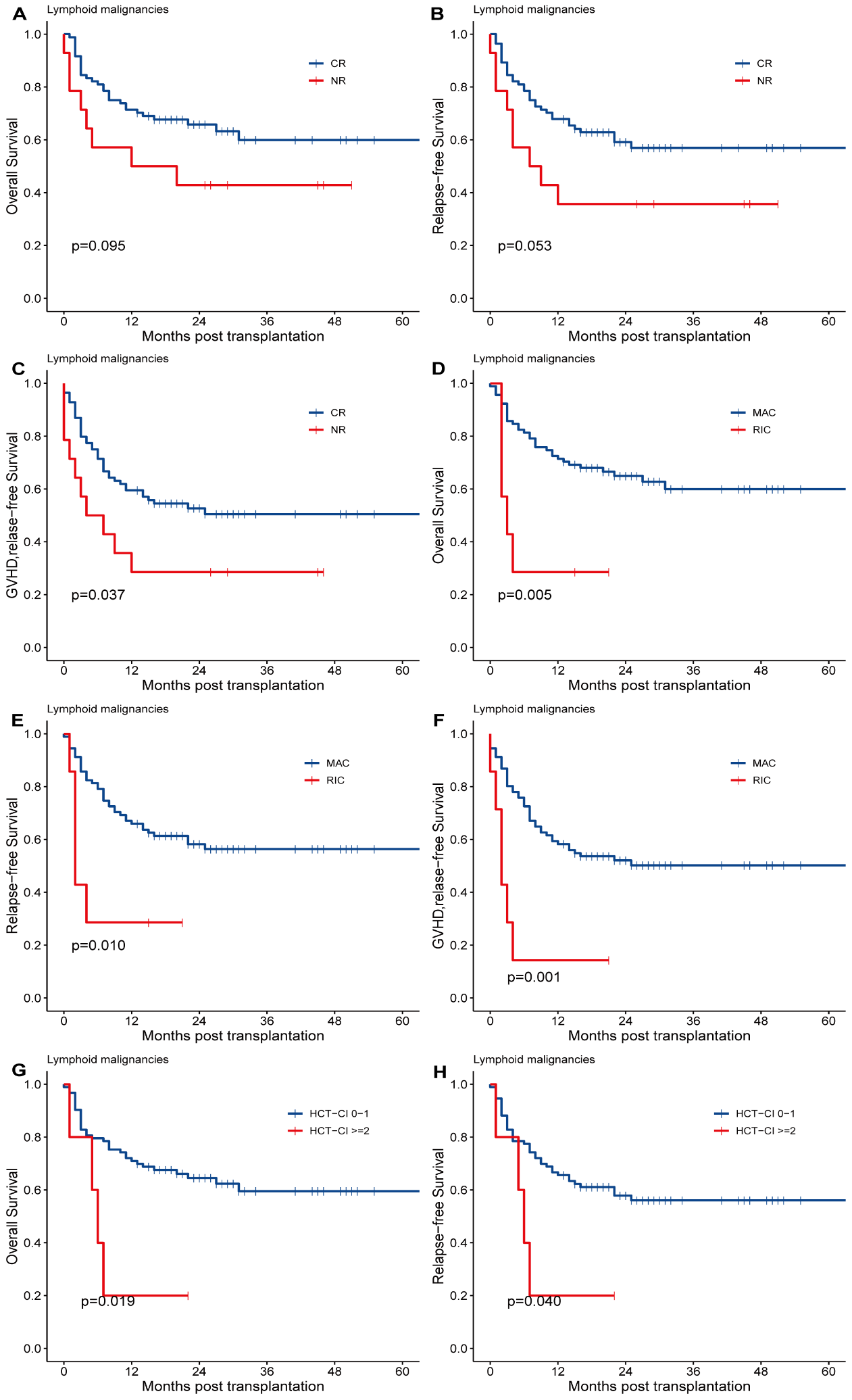


**Supplementary Figure 3.** Survival outcomes of the lymphoid malignancies. OS, RFS, and GRFS are shown for A, B, and C by disease status at transplantation, and for D, E, and F by the conditioning regimens. OS and RFS are shown for G, and H by the HCT-CI scores.

Table S1. Univariate analysis for OS of the entire cohort

| OS | p. value | HR (95% CI for HR) |
| --- | --- | --- |
| Recipient sex (female *vs* male) | 0.127 | 0.72 (0.479-1.1) |
| Recipient age (＞median age vs ≤median age) | 0.019 | 1.6 (1.08-2.34) |
| Diagnosis (lymphoid *vs* myeloid) | 0.683 | 0.92 (0.617-1.37) |
| Disease status (NR *vs* CR) | 0.000 | 2.1 (1.45-3.14) |
| Regimen (RIC *vs* MAC) | 0.028 | 1.6 (1.05-2.52) |
| ECOG (2-4 *vs* 0-1) | 0.008 | 2.3 (1.24-4.34) |
| HCT-CI (≥2 *vs* 0-1) | 0.536 | 1.2 (0.64-2.36) |
| Donor-recipient sex (female-male *vs* others) | 0.463 | 0.83 (0.497-1.37) |
| Donor age | 0.656 | 1 (0.982-1.01) |
| Donor–recipient blood type (mismatched *vs* matched) | 0.396 | 1.2 (0.804-1.73) |
| PBSC graft MNCs | 0.575 | 0.99 (0.957-1.02) |
| PBSC graft CD34^+^cells | 0.922 | 1 (0.964-1.03) |
| PBSC graft CD3^+^cells | 0.673 | 0.97 (0.85-1.11) |
| UCB (with *vs* without) | 0.804 | 0.95 (0.63-1.43) |
| UCB nucleated cells | 0.207 | 0.74 (0.462-1.18) |
| UCB CD34+ cells | 0.451 | 0.96 (0.865-1.07) |
| aGVHD (grade Ⅰ-Ⅳ *vs* grade 0) | 0.509 | 0.87 (0.581-1.31) |
| aGVHD (grade Ⅱ-Ⅳ *vs* grade 0-Ⅰ) | 0.403 | 1.3 (0.737-2.14) |
| cGVHD (with *vs* without) | 0.028 | 0.61 (0.398-0.95) |
| cGVHD (moderate/severe *vs* non/mild) | 0.517 | 0.85 (0.522-1.39) |

Table S2. Univariate analysis for RFS of the entire cohort

| RFS | p. value | HR (95% CI for HR) |
| --- | --- | --- |
| Recipient sex (female *vs* male) | 0.090 | 0.71 (0.474-1.06) |
| Recipient age (＞median age vs ≤median age) | 0.036 | 1.5 (1.03-2.16) |
| Diagnosis (lymphoid *vs* myeloid) | 0.995 | 1 (0.682-1.46) |
| Disease status (NR *vs* CR) | 0.000 | 2 (1.4-2.96) |
| Regimen (RIC *vs* MAC) | 0.059 | 1.5 (0.985-2.3) |
| ECOG (2-4 *vs* 0-1) | 0.012 | 2.2 (1.2-4.16) |
| HCT-CI (≥2 *vs* 0-1) | 0.771 | 1.1 (0.575-2.11) |
| Donor-recipient sex (female-male *vs* others) | 0.333 | 0.78 (0.478-1.28) |
| Donor age | 0.832 | 1 (0.984-1.01) |
| Donor–recipient blood type (mismatched *vs* matched) | 0.588 | 1.1 (0.765-1.6) |
| PBSC graft MNCs | 0.537 | 0.99 (0.958-1.02) |
| PBSC graft CD34^+^cells | 0.711 | 0.99 (0.96-1.03) |
| PBSC graft CD3^+^cells | 0.454 | 0.95 (0.832-1.09) |
| UCB (with *vs* without) | 0.783 | 1.1 (0.716-1.56) |
| UCB nucleated cells | 0.287 | 0.79 (0.518-1.22) |
| UCB CD34+ cells | 0.433 | 0.96 (0.87-1.06) |
| aGVHD (grade Ⅰ-Ⅳ *vs* grade 0) | 0.497 | 0.87 (0.592-1.29) |
| aGVHD (grade Ⅱ-Ⅳ *vs* grade 0-Ⅰ) | 0.324 | 1.3 (0.778-2.14) |
| cGVHD (with *vs* without) | 0.037 | 0.64 (0.424-0.974) |
| cGVHD (moderate/severe *vs* non/mild) | 0.621 | 0.89 (0.557-1.42) |

Table S3. Univariate analysis for GRFS of the entire cohort

| GRFS | p. value | HR (95% CI for HR) |
| --- | --- | --- |
| Recipient sex (female *vs* male) | 0.332 | 0.84 (0.581-1.2) |
| Recipient age (＞median age vs ≤median age) | 0.069 | 1.4 (0.975-1.94) |
| Diagnosis (lymphoid *vs* myeloid) | 0.990 | 1 (0.701-1.42) |
| Disease status (NR *vs* CR) | 0.000 | 2 (1.39-2.8) |
| Regimen (RIC *vs* MAC) | 0.064 | 1.5 (0.979-2.16) |
| ECOG (2-4 *vs* 0-1) | 0.003 | 2.4 (1.34-4.24) |
| HCT-CI (≥2 *vs* 0-1) | 0.685 | 1.1 (0.624-2.05) |
| Donor-recipient sex (female-male *vs* others) | 0.425 | 0.83 (0.53-1.31) |
| Donor age | 0.227 | 1 (0.995-1.02) |
| Donor–recipient blood type (mismatched *vs* matched) | 0.212 | 1.2 (0.883-1.76) |
| PBSC graft MNCs | 0.471 | 0.99 (0.96-1.02) |
| PBSC graft CD34^+^cells | 0.518 | 0.99 (0.958-1.02) |
| PBSC graft CD3^+^cells | 0.878 | 0.99 (0.882-1.11) |
| UCB (with *vs* without) | 0.842 | 1 (0.721-1.49) |
| UCB nucleated cells | 0.113 | 0.72 (0.473-1.08) |
| UCB CD34+ cells | 0.189 | 0.94 (0.853-1.03) |

Table S4. Univariate analysis for NRM of the entire cohort

| NRM | p. value | HR (95% CI for HR) |
| --- | --- | --- |
| Recipient sex (female *vs* male) | 0.250 | 0.733 (0.432-1.24) |
| Recipient age (＞median age vs ≤median age) | 0.000 | 2.58 (1.53-4.37) |
| Diagnosis (lymphoid *vs* myeloid) | 0.670 | 0.896(0.539-1.49) |
| Disease status (NR *vs* CR) | 0.044 | 1.68 (1.01-2.77) |
| Regimen (RIC *vs* MAC) | 0.004 | 2.14 (1.28-3.58) |
| ECOG (2-4 *vs* 0-1) | 0.040 | 2.21 (1.04-4.70) |
| HCT-CI (≥2 *vs* 0-1) | 0.350 | 1.47 (0.651-3.31) |
| Donor-recipient sex (female-male *vs* others) | 0.980 | 1.01(0.542-1.87) |
| Donor age | 0.760 | 1 (0.986-1.02) |
| Donor–recipient blood type (mismatched *vs* matched) | 0.290 | 1.31 (0.798-2.15) |
| PBSC graft MNCs | 0.310 | 0.974 (0.924-1.03) |
| PBSC graft CD34^+^cells | 0.190 | 0.967 (0.919-1.02) |
| PBSC graft CD3^+^cells | 0.620 | 1.05 (0.88-1.24) |
| UCB (with *vs* without) | 0.530 | 0.839 (0.486-1.45) |
| UCB nucleated cells | 0.120 | 0.654 (0.385-1.11) |
| UCB CD34+ cells | 0.550 | 0.962 (0.847-1.09) |
| aGVHD (grade Ⅰ-Ⅳ *vs* grade 0) | 0.530 | 1.17 (0.716-1.91) |
| aGVHD (grade Ⅱ-Ⅳ *vs* grade 0-Ⅰ) | 0.045 | 1.80 (1.01-3.2) |
| cGVHD (with *vs* without) | 0.770 | 0.93(0.565-1.53) |
| cGVHD (moderate/severe *vs* non/mild) | 0.820 | 1.07 (0.613-1.86) |

Table S5. Univariate analysis for relapse of the entire cohort

| Relapse | p. value | HR (95% CI for HR) |
| --- | --- | --- |
| Recipient sex (female *vs* male) | 0.360 | 0.758 (0.421-1.36) |
| Recipient age (＞median age vs ≤median age) | 0.022 | 0.519 (0.296-0.91) |
| Diagnosis (lymphoid *vs* myeloid) | 0.560 | 1.18 (0.678-2.04) |
| Disease status (NR *vs* CR) | 0.019 | 1.92 (1.11-3.32) |
| Regimen (RIC *vs* MAC) | 0.280 | 0.659 (0.31-1.4) |
| ECOG (2-4 *vs* 0-1) | 0.520 | 1.41 (0.491-4.06) |
| HCT-CI (≥2 *vs* 0-1) | 0.410 | 0.615 (0.192-1.97) |
| Donor-recipient sex (female-male *vs* others) | 0.210 | 0.609 (0.28-1.32) |
| Donor age | 0.530 | 0.992 (0.968-1.02) |
| Donor–recipient blood type (mismatched *vs* matched) | 0.640 | 0.877 (0.507-1.52) |
| PBSC graft MNCs | 0.560 | 1.01 (0.972-1.05) |
| PBSC graft CD34^+^cells | 0.250 | 1.03 (0.981-1.08) |
| PBSC graft CD3^+^cells | 0.12 | 0.834 (0.655-1.05) |
| UCB (with *vs* without) | 0.190 | 1.44 (0.833-2.5) |
| UCB nucleated cells | 0.990 | 1 (0.611-1.65) |
| UCB CD34+ cells | 0.710 | 0.973 (0.843-1.12) |
| aGVHD (grade Ⅰ-Ⅳ *vs* grade 0) | 0.160 | 0.642 (0.347-1.19) |
| aGVHD (grade Ⅱ-Ⅳ *vs* grade 0-Ⅰ) | 0.420 | 0.682 (0.272-1.71) |
| cGVHD (with *vs* without) | 0.049 | 0.525 (0.276-0.997) |
| cGVHD (moderate/severe *vs* non/mild) | 0.670 | 0.864 (0.437-1.71) |

Table S6. Univariate analysis for OS of myeloid malignancies

| OS | p. value | HR (95% CI for HR) |
| --- | --- | --- |
| Recipient sex (female *vs* male) | 0.045 | 0.58 (0.34-0.988) |
| Recipient age (＞median age vs ≤median age) | 0.033 | 1.7 (1.04-2.76) |
| Disease status (NR *vs* CR) | 0.001 | 2.3 (1.44-3.82) |
| Regimen (RIC *vs* MAC) | 0.187 | 1.4 (0.847-2.34) |
| ECOG (2-4 *vs* 0-1) | 0.005 | 2.5 (1.33-4.86) |
| HCT-CI (≥2 *vs* 0-1) | 0.662 | 0.83 (0.358-1.92) |
| Donor-recipient sex (female-male *vs* others) | 0.965 | 0.99 (0.547-1.78) |
| Donor age | 0.535 | 0.99 (0.977-1.01) |
| Donor–recipient blood type (mismatched *vs* matched) | 0.242 | 1.3 (0.822-2.17) |
| PBSC graft MNCs | 0.199 | 0.97 (0.93-1.02) |
| PBSC graft CD34^+^cells | 0.370 | 0.98 (0.937-1.02) |
| PBSC graft CD3^+^cells | 0.824 | 0.98 (0.84-1.15) |
| UCB (with *vs* without) | 0.320 | 1.3 (0.752-2.39) |
| UCB nucleated cells | 0.886 | 0.95 (0.473-1.91) |
| UCB CD34+ cells | 0.471 | 1.1 (0.88-1.32) |
| aGVHD (grade Ⅰ-Ⅳ *vs* grade 0) | 0.132 | 0.65 (0.373-1.14) |
| aGVHD (grade Ⅱ-Ⅳ *vs* grade 0-Ⅰ) | 0.773 | 0.89 (0.407-1.95) |
| cGVHD (with *vs* without) | 0.019 | 0.52 (0.296-0.898) |
| cGVHD (moderate/severe *vs* non/mild) | 0.302 | 0.73 (0.395-1.33) |

Table S7. Univariate analysis for RFS of myeloid malignancies

| RFS | p. value | HR (95% CI for HR) |
| --- | --- | --- |
| Recipient sex (female *vs* male) | 0.046 | 0.59 (0.349-0.989) |
| Recipient age (＞median age vs ≤median age) | 0.051 | 1.6 (0.998-2.58) |
| Disease status (NR *vs* CR) | 0.001 | 2.2 (1.39-3.6) |
| Regimen (RIC *vs* MAC) | 0.223 | 1.4 (0.828-2.24) |
| ECOG (2-4 *vs* 0-1) | 0.004 | 2.6 (1.35-4.91) |
| HCT-CI (≥2 *vs* 0-1) | 0.555 | 0.78 (0.336-1.8) |
| Donor-recipient sex (female-male *vs* others) | 0.991 | 1 (0.566-1.78) |
| Donor age | 0.520 | 0.99 (0.977-1.01) |
| Donor–recipient blood type (mismatched *vs* matched) | 0.364 | 1.2 (0.775-2) |
| PBSC graft MNCs | 0.203 | 0.97 (0.932-1.02) |
| PBSC graft CD34^+^cells | 0.452 | 0.98 (0.942-1.03) |
| PBSC graft CD3^+^cells | 0.686 | 0.97 (0.828-1.13) |
| UCB (with *vs* without) | 0.376 | 1.3 (0.73-2.3) |
| UCB nucleated cells | 0.869 | 0.94 (0.468-1.9) |
| UCB CD34+ cells | 0.660 | 1 (0.852-1.29) |
| aGVHD (grade Ⅰ-Ⅳ *vs* grade 0) | 0.074 | 0.6 (0.349-1.05) |
| aGVHD (grade Ⅱ-Ⅳ *vs* grade 0-Ⅰ) | 0.623 | 0.82 (0.376-1.8) |
| cGVHD (with *vs* without) | 0.038 | 0.57 (0.336-0.97) |
| cGVHD (moderate/severe *vs* non/mild) | 0.535 | 0.83 (0.47-1.48) |

Table S8. Univariate analysis for GRFS of myeloid malignancies

| GRFS | p. value | HR (95% CI for HR) |
| --- | --- | --- |
| Recipient sex (female *vs* male) | 0.109 | 0.68 (0.423-1.09) |
| Recipient age (＞median age vs ≤median age) | 0.033 | 1.6 (1.04-2.51) |
| Disease status (NR *vs* CR) | 0.001 | 2.1 (1.36-3.29) |
| Regimen (RIC *vs* MAC) | 0.326 | 1.3 (0.793-2.01) |
| ECOG (2-4 *vs* 0-1) | 0.010 | 2.3 (1.22-4.17) |
| HCT-CI (≥2 *vs* 0-1) | 0.814 | 0.92 (0.441-1.9) |
| Donor-recipient sex (female-male *vs* others) | 0.747 | 1.1 (0.645-1.84) |
| Donor age | 0.990 | 1 (0.984-1.02) |
| Donor–recipient blood type (mismatched *vs* matched) | 0.084 | 1.5 (0.949-2.29) |
| PBSC graft MNCs | 0.081 | 0.97 (0.928-1) |
| PBSC graft CD34^+^cells | 0.376 | 0.98 (0.944-1.02) |
| PBSC graft CD3^+^cells | 0.802 | 0.98 (0.853-1.13) |
| UCB (with *vs* without) | 0.125 | 1.5 (0.892-2.55) |
| UCB nucleated cells | 0.271 | 0.67 (0.328-1.37) |
| UCB CD34+ cells | 0.879 | 0.98 (0.811-1.2) |

Table S9. Univariate analysis for NRM of myeloid malignancies

| NRM | p. value | HR (95% CI for HR) |
| --- | --- | --- |
| Recipient sex (female *vs* male) | 0.067 | 0.52 (0.258-1.05) |
| Recipient age (＞median age vs ≤median age) | 0.003 | 2.76 (1.43-5.32) |
| Disease status (NR *vs* CR) | 0.030 | 1.97 (1.07-3.64) |
| Regimen (RIC *vs* MAC) | 0.013 | 2.16 (1.18-3.99) |
| ECOG (2-4 *vs* 0-1) | 0.028 | 2.36 (1.10-5.08) |
| HCT-CI (≥2 *vs* 0-1) | 0.950 | 0.968 (0.326-2.88) |
| Donor-recipient sex (female-male *vs* others) | 0.480 | 1.29 (0.64-2.6) |
| Donor age | 0.490 | 1.01 (0.988-1.03) |
| Donor–recipient blood type (mismatched *vs* matched) | 0.420 | 1.29 (0.695-2.39) |
| PBSC graft MNCs | 0.130 | 0.949 (0.887-1.02) |
| PBSC graft CD34^+^cells | 0.072 | 0.947 (0.892-1) |
| PBSC graft CD3^+^cells | 0.550 | 1.06 (0.873-1.29) |
| UCB (with *vs* without) | 0.640 | 1.20 (0.553-2.62) |
| UCB nucleated cells | 0.410 | 0.715 (0.322-1.59) |
| UCB CD34+ cells | 0.600 | 1.08 (0.814-1.43) |
| aGVHD (grade Ⅰ-Ⅳ *vs* grade 0) | 0.480 | 0.791 (0.411-1.52) |
| aGVHD (grade Ⅱ-Ⅳ *vs* grade 0-Ⅰ) | 0.870 | 1.08 (0.443-2.62) |
| cGVHD (with *vs* without) | 0.390 | 0.761 (0.405-1.43) |
| cGVHD (moderate/severe *vs* non/mild) | 0.970 | 0.988 (0.505-1.94) |

Table S10. Univariate analysis for relapse of myeloid malignancies

| Relapse | p. value | HR (95% CI for HR) |
| --- | --- | --- |
| Recipient sex (female *vs* male) | 0.590 | 0.813 (0.382-1.73) |
| Recipient age (＞median age vs ≤median age) | 0.200 | 0.618 (0.295-1.29) |
| Disease status (NR *vs* CR) | 0.066 | 1.95 (0.957-3.97) |
| Regimen (RIC *vs* MAC) | 0.170 | 0.541 (0.223-1.31) |
| ECOG (2-4 *vs* 0-1) | 0.350 | 1.68 (0.568-5.0) |
| HCT-CI (≥2 *vs* 0-1) | 0.400 | 0.541 (0.128-2.29) |
| Donor-recipient sex (female-male *vs* others) | 0.420 | 0.684 (0.269-1.74) |
| Donor age | 0.150 | 0.976 (0.944-1.01) |
| Donor–recipient blood type (mismatched *vs* matched) | 0.860 | 1.07 (0.523-2.18) |
| PBSC graft MNCs | 0.690 | 1.01 (0.957-1.07) |
| PBSC graft CD34^+^cells | 0.260 | 1.03 (0.976-1.09) |
| PBSC graft CD3^+^cells | 0.170 | 0.824 (0.626-1.08) |
| UCB (with *vs* without) | 0.440 | 1.38 (0.607-3.16) |
| UCB nucleated cells | 0.610 | 1.25 (0.531-2.96) |
| UCB CD34+ cells | 0.910 | 0.982 (0.724-1.33) |
| aGVHD (grade Ⅰ-Ⅳ *vs* grade 0) | 0.140 | 0.517 (0.213-1.25) |
| aGVHD (grade Ⅱ-Ⅳ *vs* grade 0-Ⅰ) | 0.400 | 0.534 (0.122-2.33) |
| cGVHD (with *vs* without) | 0.120 | 0.53 (0.238-1.18) |
| cGVHD (moderate/severe *vs* non/mild) | 0.680 | 0.841 (0.366-1.93) |

Table S11. Univariate analysis for OS of lymphoid malignancies

| OS | p. value | HR (95% CI for HR) |
| --- | --- | --- |
| Recipient sex (female *vs* male) | 0.877 | 1.1 (0.545-2.04) |
| Recipient age (＞median age vs ≤median age) | 0.714 | 0.89 (0.469-1.68) |
| Disease status (NR *vs* CR) | 0.095 | 1.9 (0.891-4.25) |
| Regimen (RIC *vs* MAC) | 0.005 | 3.9 (1.5-10.2) |
| HCT-CI (≥2 *vs* 0-1) | 0.019 | 3.5 (1.22-10) |
| Donor-recipient sex (female-male *vs* others) | 0.202 | 0.51 (0.18-1.44) |
| Donor age | 0.794 | 1 (0.979-1.03) |
| Donor–recipient blood type (mismatched *vs* matched) | 0.864 | 0.95 (0.499-1.79) |
| PBSC graft MNCs | 0.393 | 1 (0.97-1.08) |
| PBSC graft CD34^+^cells | 0.252 | 1 (0.977-1.09) |
| PBSC graft CD3^+^cells | 0.610 | 0.94 (0.728-1.2) |
| UCB (with *vs* without) | 0.246 | 0.69 (0.363-1.3) |
| UCB nucleated cells | 0.200 | 0.65 (0.342-1.25) |
| UCB CD34+ cells | 0.402 | 0.94 (0.819-1.08) |
| aGVHD (grade Ⅰ-Ⅳ *vs* grade 0) | 0.339 | 1.4 (0.722-2.58) |
| aGVHD (grade Ⅱ-Ⅳ *vs* grade 0-Ⅰ) | 0.173 | 1.7 (0.788-3.78) |
| cGVHD (with *vs* without) | 0.580 | 0.82 (0.406-1.66) |
| cGVHD (moderate/severe *vs* non/mild) | 0.770 | 1.1 (0.497-2.57) |

Table S12. Univariate analysis for RFS of lymphoid malignancies

| RFS | p. value | HR (95% CI for HR) |
| --- | --- | --- |
| Recipient sex (female *vs* male) | 0.889 | 0.96 (0.511-1.79) |
| Recipient age (＞median age vs ≤median age) | 0.531 | 0.83 (0.453-1.5) |
| Disease status (NR *vs* CR) | 0.053 | 2.1 (0.99-4.32) |
| Regimen (RIC *vs* MAC) | 0.010 | 3.5 (1.35-8.96) |
| HCT-CI (≥2 *vs* 0-1) | 0.040 | 3 (1.05-8.38) |
| Donor-recipient sex (female-male *vs* others) | 0.116 | 0.44 (0.156-1.23) |
| Donor age | 0.477 | 1 (0.985-1.03) |
| Donor–recipient blood type (mismatched *vs* matched) | 0.741 | 0.9 (0.495-1.65) |
| PBSC graft MNCs | 0.473 | 1 (0.968-1.07) |
| PBSC graft CD34^+^cells | 0.648 | 1 (0.958-1.07) |
| PBSC graft CD3^+^cells | 0.418 | 0.91 (0.712-1.15) |
| UCB (with *vs* without) | 0.655 | 0.87 (0.477-1.59) |
| UCB nucleated cells | 0.272 | 0.73 (0.423-1.27) |
| UCB CD34+ cells | 0.342 | 0.94 (0.831-1.07) |
| aGVHD (grade Ⅰ-Ⅳ *vs* grade 0) | 0.241 | 1.4 (0.786-2.6) |
| aGVHD (grade Ⅱ-Ⅳ *vs* grade 0-Ⅰ) | 0.082 | 1.9 (0.924-3.81) |
| cGVHD (with *vs* without) | 0.495 | 0.79 (0.407-1.54) |
| cGVHD (moderate/severe *vs* non/mild) | 0.938 | 1 (0.459-2.32) |

Table S13. Univariate analysis for GRFS of lymphoid malignancies

| GRFS | p. value | HR (95% CI for HR) |
| --- | --- | --- |
| Recipient sex (female *vs* male) | 0.590 | 1.2 (0.661-2.07) |
| Recipient age (＞median age vs ≤median age) | 0.273 | 0.73 (0.418-1.28) |
| Disease status (NR *vs* CR) | 0.037 | 2.1 (1.05-4.19) |
| Regimen (RIC *vs* MAC) | 0.001 | 4.3 (1.78-10.2) |
| HCT-CI (≥2 *vs* 0-1) | 0.131 | 2.2 (0.79-6.19) |
| Donor-recipient sex (female-male *vs* others) | 0.091 | 0.45 (0.179-1.14) |
| Donor age | 0.131 | 1 (0.995-1.04) |
| Donor–recipient blood type (mismatched *vs* matched) | 0.803 | 0.93 (0.534-1.63) |
| PBSC graft MNCs | 0.244 | 1 (0.982-1.08) |
| PBSC graft CD34^+^cells | 0.845 | 1 (0.953-1.06) |
| PBSC graft CD3^+^cells | 0.905 | 1 (0.818-1.25) |
| UCB (with *vs* without) | 0.228 | 0.71 (0.407-1.24) |
| UCB nucleated cells | 0.293 | 0.76 (0.453-1.27) |
| UCB CD34+ cells | 0.312 | 0.94 (0.836-1.06) |

Table S14. Univariate analysis for NRM of lymphoid malignancies

| NRM | p. value | HR (95% CI for HR) |
| --- | --- | --- |
| Recipient sex (female *vs* male) | 0.550 | 1.29 (0.561-2.97) |
| Recipient age (＞median age vs ≤median age) | 0.340 | 1.51 (0.654-3.48) |
| Disease status (NR *vs* CR) | 0.960 | 1.04 (0.281-3.84) |
| Regimen (RIC *vs* MAC) | 0.130 | 2.54 (0.763-8.44) |
| HCT-CI (≥2 *vs* 0-1) | 0.023 | 3.82 (1.20-12.20) |
| Donor-recipient sex (female-male *vs* others) | 0.310 | 0.478 (0.115-1.98) |
| Donor age | 0.390 | 0.986 (0.954-1.02) |
| Donor–recipient blood type (mismatched *vs* matched) | 0.480 | 1.35 (0.593-3.07) |
| PBSC graft MNCs | 0.660 | 1.02 (0.941-1.10) |
| PBSC graft CD34^+^cells | 0.930 | 1.00 (0.923-1.09) |
| PBSC graft CD3^+^cells | 0.920 | 0.984 (0.727-1.33) |
| UCB (with *vs* without) | 0.220 | 0.598 (0.262-1.36) |
| UCB nucleated cells | 0.240 | 0.654 (0.321-1.33) |
| UCB CD34+ cells | 0.370 | 0.936 (0.811-1.08) |
| aGVHD (grade Ⅰ-Ⅳ *vs* grade 0) | 0.059 | 2.29 (0.968-5.41) |
| aGVHD (grade Ⅱ-Ⅳ *vs* grade 0-Ⅰ) | 0.016 | 2.84 (1.22-6.61) |
| cGVHD (with *vs* without) | 0.530 | 1.30 (0.576-2.91) |
| cGVHD (moderate/severe *vs* non/mild) | 0.690 | 1.22 (0.457-3.26) |

Table S15. Univariate analysis for relapse of lymphoid malignancies

| Relapse | p. value | HR (95% CI for HR) |
| --- | --- | --- |
| Recipient sex (female *vs* male) | 0.420 | 0.684 (0.271-1.73) |
| Recipient age (＞median age vs ≤median age) | 0.031 | 0.355 (0.139-0.909) |
| Disease status (NR *vs* CR) | 0.036 | 2.74 (1.07-7.02) |
| Regimen (RIC *vs* MAC) | 0.500 | 1.74 (0.348-8.76) |
| HCT-CI (≥2 *vs* 0-1) | 0.960 | 0.945 (0.127-7.05) |
| Donor-recipient sex (female-male *vs* others) | 0.350 | 0.503 (0.12-2.11) |
| Donor age | 0.085 | 1.03 (0.996-1.07) |
| Donor–recipient blood type (mismatched *vs* matched) | 0.320 | 0.649 (0.277-1.52) |
| PBSC graft MNCs | 0.660 | 1.01 (0.954-1.08) |
| PBSC graft CD34^+^cells | 0.660 | 1.02 (0.937-1.11) |
| PBSC graft CD3^+^cells | 0.450 | 0.859 (0.578-1.28) |
| UCB (with *vs* without) | 0.380 | 1.50 (0.605-3.72) |
| UCB nucleated cells | 0.760 | 0.904 (0.478-1.71) |
| UCB CD34+ cells | 0.660 | 0.962 (0.811-1.14) |
| aGVHD (grade Ⅰ-Ⅳ *vs* grade 0) | 0.530 | 0.757 (0.315-1.82) |
| aGVHD (grade Ⅱ-Ⅳ *vs* grade 0-Ⅰ) | 0.830 | 0.878 (0.274-2.81) |
| cGVHD (with *vs* without) | 0.250 | 0.531 (0.18-1.56) |
| cGVHD (moderate/severe *vs* non/mild) | 0.950 | 0.965 (0.288-3.23) |
